# Clinical efficacy of probiotic supplementation in the treatment of knee osteoarthritis: a meta-analysis

**DOI:** 10.3389/fmicb.2025.1526690

**Published:** 2025-04-09

**Authors:** Miao Tian, Youyang Zhu, Shiyu Lu, Yuliang Qin, Xinyao Li, Tao Wang, Ying Guo, Hongling Shi, Dongdong Qin

**Affiliations:** ^1^Kunming Municipal Hospital of Traditional Chinese Medicine, Third Affiliated Hospital, Yunnan University of Chinese Medicine, Kunming, Yunnan, China; ^2^First Clinical Medical College, Yunnan University of Chinese Medicine, Kunming, Yunnan, China; ^3^The People’s Hospital of Mengzi, The Affiliated Hospital of Yunnan University of Chinese Medicine, Mengzi, Honghe, China; ^4^Key Laboratory of Traditional Chinese Medicine for Prevention and Treatment of Neuropsychiatric Diseases, Yunnan University of Chinese Medicine, Kunming, Yunnan, China; ^5^Department of Rehabilitation Medicine, The Third People’s Hospital of Yunnan Province, Kunming, Yunnan, China

**Keywords:** knee osteoarthritis, probiotics, clinical efficacy, gut-joint axis, systematic evaluation

## Abstract

**Background:**

We aimed to systematically evaluate and analyze the clinical efficacy of oral probiotics in the treatment of knee osteoarthritis (KOA) based on the theory of “gut-joint axis.”

**Methods:**

We searched PubMed, The Cochrane Library, Embase, China Knowledge Network (CNKI), Wanfang Database, and Wipro Database (CQVIP) databases for clinical randomized controlled trials of oral probiotics for the treatment of KOA. The literature was organized by Note express software, and the quality of the included literature was evaluated according to the Cochrane systematic evaluation method, and meta-analysis was performed using RevMan 5.4 software.

**Results:**

Five randomized controlled trials with 694 participants were included in this study, and the results of the meta-analysis showed that the observation group experienced significant reductions in the Western Ontario and McMaster Universities Osteoarthritis Index total score, visual analog score, and high-sensitivity C-reactive protein level compared to the control group, but did not show significant differences in improvement of stiffness and regulation of body weight.

**Conclusion:**

Oral probiotics had an ameliorative effect on function, pain, and inflammatory response in patients with KOA, but our results need to be validated in future large-scale studies.

**Systematic review registration:**

The website is https://www.crd.york.ac.uk/PROSPERO/.

## 1 Introduction

Osteoarthritis (OA) represents a significant degenerative musculoskeletal disorder characterized by articular cartilage deterioration, synovial inflammation, and structural alterations in the osteoarticular complex (Hunter and Bierma-Zeinstra, 2019; Leifer et al., 2022; Yue and Berman, 2022). Global epidemiological data indicates a 15% prevalence among individuals aged 30 years and above, with projections suggesting an affected population of approximately 1 billion by 2050 (GBD 2021 Osteoarthritis Collaborators, 2023). The knee joint, due to its weight-bearing function and intricate biomechanical architecture, demonstrates particular susceptibility to osteoarthritic degeneration. Contemporary demographic shifts, including increased longevity, altered nutritional patterns, and rising obesity rates, have contributed to the escalating incidence of knee osteoarthritis (KOA), necessitating enhanced focus on preventive and therapeutic interventions (Ferreira et al., 2018). The pathophysiological mechanisms underlying KOA are multifaceted, encompassing chronic inflammatory cascades, chondral degeneration, and metabolic dysregulation, with inflammatory processes being particularly significant in disease progression (Chisari et al., 2021; Wei et al., 2022). Recent advances in microbiome research have illuminated the crucial role of gut microbial homeostasis in arthritic conditions, establishing the “gut-joint axis” paradigm (Cypers et al., 2014; Ulici et al., 2018). This biological framework elucidates the bidirectional relationship between gastrointestinal and articular health, whereby perturbations in gut microbiota can precipitate joint inflammation through immune-mediated bacterial translocation to synovial tissues, and conversely, joint pathology may influence gut health (Brandtzaeg, 1997; Biver et al., 2019).

Research indicates the human gut harbors approximately 1 × 10^14^ microorganisms encompassing 1,000–1,150 distinct species, with predominant colonization in the colonic region, rendering the intestinal microbiota a significant area of scientific investigation (Qin et al., 2010). The host organism provides an optimal microenvironment and nutritional substrates for these microorganisms, while the microbiota reciprocally facilitates host metabolic development and intestinal immunological maturation through vitamin biosynthesis and short chain fatty acids production (Kau et al., 2011). The gastrointestinal tract, housing numerous innate and adaptive immune cells, represents the body’s primary immunological organ (Kamada and Núñez, 2014). The homeostatic relationship between microbiota and intestinal immunity is vital for maintaining mucosal equilibrium. Dysbiosis or immune dysregulation may precipitate various systemic inflammatory conditions, including osteoarthritic manifestations (Tajik et al., 2020). Through the lens of the gut-joint axis paradigm, therapeutic modulation of gut microbiota to ameliorate low-grade inflammation presents a promising avenue for KOA rehabilitation, potentially establishing a novel therapeutic target for both prophylaxis and treatment of KOA.

Modulation of the gut microbiome and intestinal immune system has demonstrated significant therapeutic potential in osteoarthritis management. In previous clinical and preclinical investigations, oral probiotic supplements have exhibited analgesic properties and mediates inflammatory responses (Wei et al., 2023). Intake of probiotic complex has been found reduce the levels of pro-inflammatory mediators, specifically interleukin-6 and tumor necrosis factor-alpha (TNF-*α*), while elevating anti-inflammatory IL-10 levels, resulting in attenuated inflammation, reduced cartilage degradation, and decreased nociceptive responses in osteoarthritic rodent models (Kwon et al., 2018). Administration of TCI633 in experimental KOA models demonstrated reduced chondrocyte apoptosis and ameliorated joint inflammation, including decreased synovitis and edema (Lin et al., 2021). Probiotic supplementation facilitates intestinal microenvironment homeostasis, promotes beneficial bacterial proliferation, and modulates immune responses, showing particular efficacy in immune-mediated and metabolic disorders. Furthermore, microbiome modulation represents an effective strategy for bone metabolism regulation and skeletal health optimization. Enhancement of beneficial gut microbiota can generate bacterial metabolites that modulate inflammatory markers, pain perception, and functional outcomes (Chandrasekaran et al., 2024). While probiotic supplementation presents a promising therapeutic avenue for KOA management and may serve as a valuable adjunctive therapy (Tu et al., 2021; Rahman et al., 2023), comprehensive systematic reviews evaluating clinical efficacy remain insufficient.

Consequently, this investigation involved a comprehensive literature review and meta-analytical data collection to evaluate the therapeutic efficacy, safety profile, and hematological parameter modifications associated with oral probiotic interventions in KOA management, aiming to provide evidence-based insights for clinical applications and treatment protocols.

## 2 Materials and methods

### 2.1 Inclusion and exclusion criteria

#### 2.1.1 Inclusion criteria

This investigation employed a randomized controlled trial (RCT) methodology. Study participants comprised individuals with confirmed KOA, diagnosed through comprehensive assessment including physical examination, symptomatic presentation, radiological findings, and additional diagnostic criteria. The experimental cohort received probiotic intervention, either as monotherapy or in combination with other therapeutic modalities, irrespective of probiotic strain specificity; the control cohort underwent treatment protocols excluding probiotic supplementation. Outcome measures encompassed the Western Ontario and McMaster Universities Osteoarthritis Index (WOMAC), Visual Analog Scale (VAS) for pain assessment, serum high-sensitivity C-reactive protein (hs-CRP) concentrations, inflammatory biomarkers, bone mineral density measurements, and body mass index (BMI) calculations.

#### 2.1.2 Exclusion criteria

The following articles were excluded: Identical articles; literature reviews, case-test summaries, experience sharing, and conference papers; non-clinical studies, non-randomized controlled studies, studies with subgroups larger than two, and animal studies; studies with incomplete study data; studies with an unclear diagnosis; and studies in which the probiotic intervention was not used in the observation group.

### 2.2 Literature search methods

The PubMed, Embase, the Cochrane Library, China Knowledge Network (CNKI), Wanfang Database, and CQVIP databases were searched extensively for articles on probiotics for the treatment of KOA using a computerized search from the time of database construction to July 2024. The search was conducted using a combination of subject terms and free words: “osteoarthritis,” “probiotics,” “*Streptococcus thermophilus*,” “*Bifidobacterium*,” “*Lactococcus*,” “*Bacillus subtilis*,” “*Enterococcus*,” “*Saccharomyces*” and others. Language was limited to English and Chinese.

### 2.3 Literature selection

A systematic deduplication process was conducted utilizing the Endnote software, followed by independent screening by two investigators who reviewed titles and abstracts according to predefined inclusion criteria. Articles that failed to meet the study requirements were excluded during initial screening. The remaining articles underwent full-text evaluation for secondary screening. In cases of discrepancy, a third investigator was consulted for arbitration until consensus was achieved. The final selection comprised articles that fully satisfied the study inclusion criteria.

### 2.4 Literature quality evaluation and data extraction

A systematic quality assessment was conducted utilizing the Cochrane Risk of Bias Assessment Framework to evaluate methodological rigor and potential biases in the selected studies. The evaluation encompassed critical parameters including randomization protocols, allocation concealment mechanisms, blinding procedures, reporting bias, outcome data integrity, and additional bias sources. Data extraction was systematically performed, documenting geographical origin, primary investigator, publication chronology, cohort dimensions, therapeutic interventions, treatment duration, and clinical outcomes. For studies presenting data exclusively through graphical representations, quantitative data extraction was facilitated through GetData software implementation.

### 2.5 Statistical methods

A comprehensive meta-analysis was conducted utilizing RevMan 5.4 software for data synthesis. Effect sizes were calculated using standardized mean differences (SMD) for WOMAC scores, VAS scores, and hs-CRP levels, while weighted mean difference (WMD) was employed for BMI measurements, with corresponding 95% confidence intervals. Statistical heterogeneity was assessed through *p*-values and *I*^2^ statistics. Studies were analyzed using a fixed-effects model when *p* ≥ 0.10 and *I^2^* ≤ 50%, whereas a random-effects model was applied when *p* < 0.10 and *I^2^* > 50%. In cases of significant heterogeneity, sensitivity analyses were performed through sequential study exclusion to identify heterogeneity sources and conduct further analyses. Given the limited number of included studies, funnel plot analysis for publication bias assessment was deemed statistically inappropriate and, therefore, not performed.

## 3 Results

### 3.1 Results of literature screening

**Figure 1:**
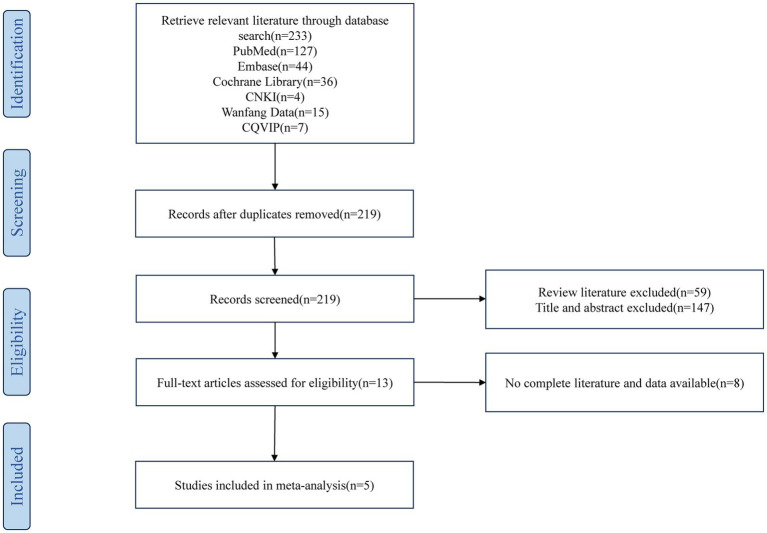
Literature screening process.

Through a systematic literature search, a total of 233 publications were initially identified. After eliminating 14 duplicate entries, 219 unique documents remained. Subsequently, 59 review articles were excluded, and an additional 147 articles were eliminated based on title and abstract screening. Further full-text assessment led to the exclusion of 7 more articles. Ultimately, 5 articles meeting the inclusion criteria were selected for analysis, as depicted in Figure 1.

### 3.2 Basic characteristics of the included literature

Upon analysis of five selected publications (Lei et al., 2017; Lyu et al., 2020; Wang, 2022; Han, 2023; Dolatkhah et al., 2024), baseline demographic characteristics were comparable between intervention and control cohorts. Dolatkhah et al. (2024) reported five cases of minor adverse events. Two participants in the experimental group experienced diarrhea and stomach pain, while three participants in the control group reported constipation, nausea and heartburn. However, no clinically significant differences were observed between the two groups. Notably, no severe adverse events were documented across all five investigations. Detailed baseline characteristics of the included studies are summarized in Table 1.

**Table 1:** General characteristics of included literature.

Author	Year	Sample size		Intervention measures		Treatment period	Outcomes reported
		Experimental	Control	Experimental	Control		
M. Lei et al.	2017	215	218	*Lactobacillus casei* Shirota	Placebo	6 months	①②③
Jia-Ling Lyu et al.	2020	37	30	TCI633	Placebo	12 weeks	①②
Hanbo Wang et al.	2022	37	28	*Bifidobacterium Lactis* + calcium Tablet, Chondroitin sulfate	Placebo + calcium tablet, Chondroitin sulfate	4 months	②④⑤
Tieling Han et al.	2023	32	34	Bifidobacterium + Glucosamine tablets	Placebo + Glucosamine tablets	3 months	②③④⑥
Neda Dolatkhah et al.	2024	32	31	Probiotics (S. boulardii) + Physical therapy, rehabilitation	Placebo + Physical therapy, rehabilitation	12 weeks	①②③⑥

①hs-CRP; ②WOMAC; ③VAS; ④Inflammatory factors; ⑤Bone density; ⑥BMI.

### 3.3 Results of risk of bias evaluation of included studies

**Figure 2:**
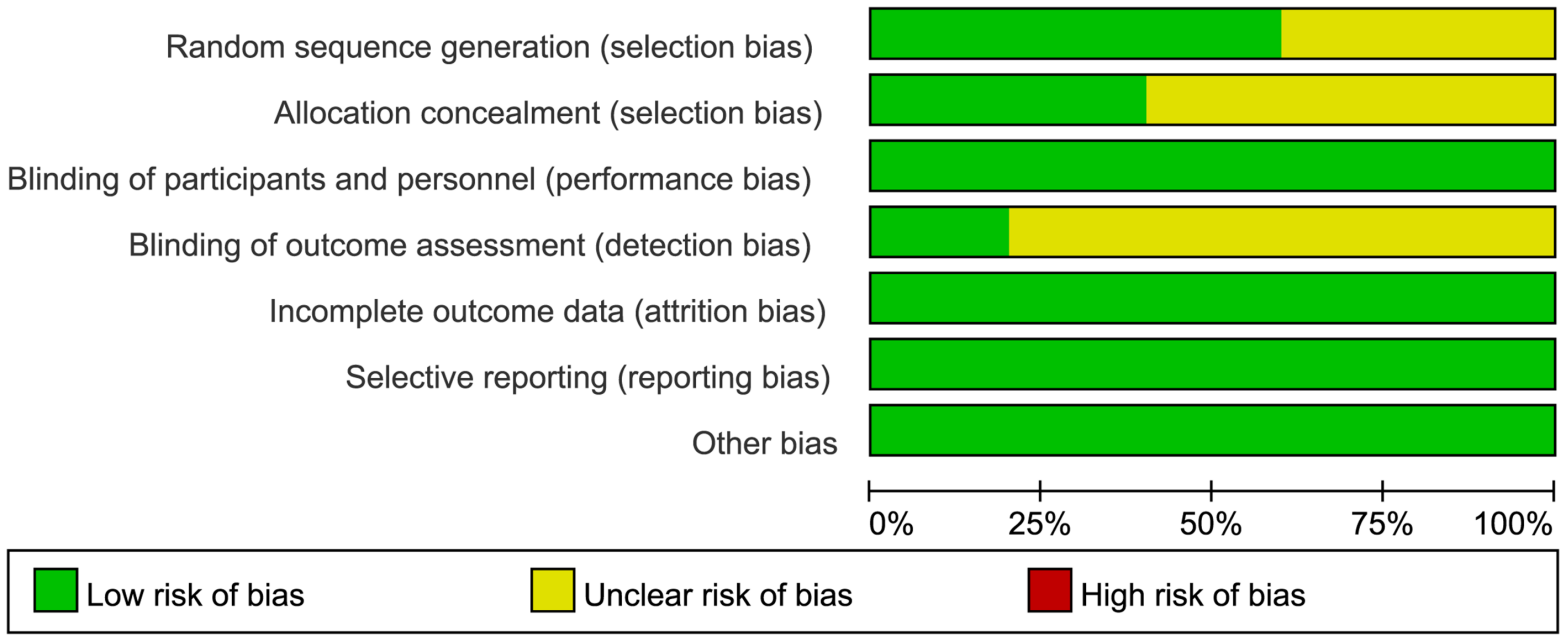
Bias risk assessment of included literature.

The analyzed literature comprised five randomized controlled trials (RCTs), with varying methodological rigor in their randomization procedures. One trial implemented computerized randomization software for allocation, while another employed a random number table methodology. Block randomization was utilized in one study, whereas two trials failed to specify their randomization protocols. Notably, three studies demonstrated methodological limitations, lacking documentation of allocation concealment and outcome assessment blinding. All five investigations provided comprehensive documentation of participant attrition, including detailed analyses of exclusion rationales. The systematic assessment of bias risk across the included studies is visually represented in Figure 2.

### 3.4 Meta-analysis results

#### 3.4.1 WOMAC score

**Figure 3:**
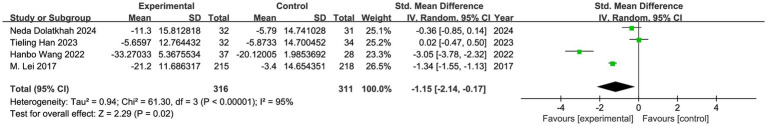
Meta-analysis of WOMAC scores in two groups of patients.

The WOMAC scores were evaluated across five studies, with detailed scores available in four publications (Lei et al., 2017; Wang, 2022; Han, 2023; Dolatkhah et al., 2024). Meta-analysis demonstrated that oral probiotic intervention significantly reduced overall WOMAC scores compared to controls (*SMD* = −1.15, 95%CI = −2.14 to −0.17, *p* = 0.002). Subgroup analysis of WOMAC components from two studies revealed no significant improvement in joint stiffness (*SMD* = −0.06, 95%CI = −0.23–0.12, *p* = 0.53), while physical function scores showed significant enhancement (*SMD* = −1.07, 95%CI = −1.26 to −0.88, *p* < 0.00001), indicating substantial functional improvement with probiotic treatment (Figure 3).

#### 3.4.2 VAS score

**Figure 4:**
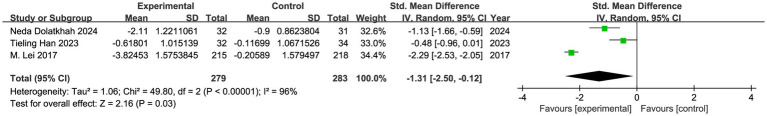
Meta-analysis of VAS scores in two groups of patients.

Three of the included papers (Lei et al., 2017; Han, 2023; Dolatkhah et al., 2024) reported VAS scores, and the meta-analysis showed that patients taking oral probiotics had a significantly better mitigating effect than the control group in reducing the VAS scores (*SMD* = −1.31, 95%CI = −2.50 to −0.12, *p* = 0.03), with a statistically significant difference (Figure 4).

#### 3.4.3 hs-CRP

**Figure 5:**
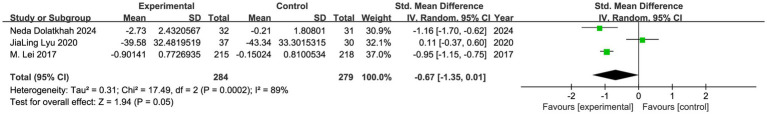
Meta-analysis of hs-CRP in two groups of patients.

Three of the included papers (Lei et al., 2017; Lyu et al., 2020; Dolatkhah et al., 2024) reported the reduction of hs-CRP, and meta-analysis showed that there was no statistically significant reduction in the hs-CRP level of patients taking oral probiotics compared to those not taking oral probiotics (*SMD* = −0.67, 95%CI = −1.35–0.01, *p* = 0.05), and the difference was not statistically significant, but the heterogeneity was high (Figure 5).

#### 3.4.4 BMI

**Figure 6:**
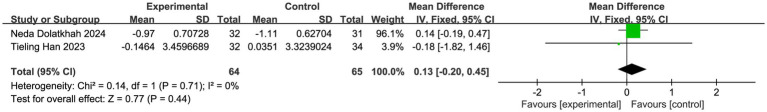
Meta-analysis of BMI in two groups of patients.

Only two publications (Han, 2023; Dolatkhah et al., 2024) reported a comparison of BMI before and after treatment, which were analyzed to show that oral probiotics did not lead to a clear impact in reducing the BMI (*WMD* = 0.13, 95%CI = −0.20–0.45, *p* = 0.44), and the difference was not statistically significant (Figure 6).

### 3.5 Heterogeneity analysis

The meta-analysis of the included literature revealed heterogeneity in the results of WOMAC score, VAS score, and hs-CRP levels. Regarding the WOMAC scores, the reanalysis after exclusion of two papers by Wang (2022) and Lei et al. (2017) revealed a decrease in heterogeneity (*p* = 0.29, *I^2^* = 9%). After reading the full article, it was found that in Wang (2022)’s study, the use of chondroitin sulfur and calcium tablets in conjunction with oral probiotics may have led to a more pronounced reduction in WOMAC scores; meanwhile, Lei et al. (2017)’s study was conducted over a 6-month period, and the long duration of treatment may have led to a more significant reduction in patients’ WOMAC scores. Further, the hs-CRP levels of patients after oral probiotic supplementation showed a significant reduction (*SMD* = −0.97, 95% CI = −1.16 to −0.79, *p* < 0.00001), and the heterogeneity was significantly reduced (*p* = 0.47, *I^2^* = 0%) after elimination of Lyu et al. (2020)’s study, suggesting that oral probiotics had a clear reduction effect on hs-CRP in KOA patients. Upon review of Lyu et al. (2020)’s study, a methodological limitation was identified in the randomization process, where the Kellgren–Lawrence (K/L) grade stratification of KOA patients was not adequately balanced between the intervention and control groups. This imbalanced distribution of disease severity potentially confounded the treatment outcomes, resulting in non-significant or inferior results in the intervention group compared to in the control group. Subsequent sensitivity analysis of VAS scores through sequential literature exclusion revealed no significant heterogeneity.

## 4 Discussions

KOA represents a multifaceted degenerative joint condition, primarily manifesting through articular pain, joint rigidity, muscular deterioration, and compromised joint functionality (Sharma, 2021). The global burden of KOA has reached epidemic proportions, affecting approximately 240 million individuals worldwide, with prevalence rates showing an upward trajectory amid demographic aging trends (Katz et al., 2021; Long et al., 2022). While conventional therapeutic approaches encompass conservative management strategies, including exercise-based interventions, physiotherapy, pharmacological treatments (both systemic and topical), and surgical procedures (Geng et al., 2023; Pires et al., 2024), their inherent limitations have prompted extensive research into alternative therapeutic modalities. Recent scientific investigations have illuminated the crucial role of intestinal immunological homeostasis in systemic health. Perturbations in the gut immune milieu can precipitate dysregulation of systemic homeostasis, triggering chronic inflammatory cascades that contribute to various extraintestinal pathologies. Emerging evidence suggests that alterations in the gut microbiome can both directly and indirectly influence musculoskeletal joint pathophysiology and accelerate osteoarthritic progression (Gilat et al., 2024). Notably, studies have documented significant reductions in gut microbial diversity among osteoarthritis patients (Favazzo et al., 2020; Lu et al., 2020; Wang et al., 2021), establishing a compelling link between intestinal dysbiosis and osteoarthritic pathogenesis (de Sire et al., 2020; Longo et al., 2024). Experimental studies, both *in vitro* and *in vivo*, have demonstrated that probiotic intervention can modulate the intestinal microbiota, resulting in enhanced musculoskeletal function and amelioration of osteoarthritic manifestations in murine models, while also attenuating IL-1-mediated chondrocyte alterations (Amin et al., 2024). The functionality of probiotics exhibits a high degree of strain specificity, and over 500 strains are currently utilized in commercially available probiotic products worldwide (Binda et al., 2020). This meta-analysis primarily investigated the following species: *Saccharomyces boulardii (S. boulardii)*, *Lactobacillus casei Shirota (LcS), Streptococcus thermophilus* (TCI633), and *Bifidobacterium*. *S. boulardii* exhibits antioxidant, antibacterial, antitumor, and anti-inflammatory properties, which can maintain the integrity of the intestinal mucosa and joints in Collagen-induced arthritis (CIA) rats, reduce pro-inflammatory cytokine levels, and modulate the gut microbiota (Fu et al., 2023; Ahmad Zhang et al., 2024). *LcS* exhibits immunomodulatory properties that enhance both innate and adaptive immune responses, playing a crucial role in the regulation of inflammatory reactions. *LcS* can alleviate the symptoms of CIA rat by increasing the proportion of Treg cells in mesenteric lymph nodes, modulating gut microbiota structure, and regulating plasma metabolites (Fan et al., 2021). It also reduces the expression of IL-1β, IL-2, and IL-6 in the serum of OA mice and inhibits cartilage degradation (So et al., 2011). TCI633 exhibits specific efficacy in modulating immune responses and alleviating metabolic diseases. It can upregulate the expression of type II collagen in the cartilage of anterior cruciate ligament transection-induced OA rat, thereby mitigating chondrocyte damage, reducing joint swelling and synovial inflammation, and also exerting an inhibitory effect on pain (Lin et al., 2021). In *Bifidobacterium* strains, *Bifidobacterium breve*, *Bifidobacterium longum*, and *Bifidobacterium animalis* can modulate the gut microbiota in OA rats, reduce the expression of the inflammatory cytokine monocyte chemoattractant protein-1 and related inflammatory factors in osteoblasts, thereby alleviating the symptoms of osteoarthritis (Li et al., 2022; Yang et al., 2025; Oh et al., 2023). However, the clinical efficacy of exogenous microbial supplementation in KOA management has shown variable outcomes. This meta-analysis aims to evaluate the therapeutic efficacy of oral probiotic supplementation in clinical KOA management, guided by the gut-joint axis paradigm.

Based on systematic review and meta-analytical findings, oral probiotic supplementation demonstrated superior efficacy compared to placebo controls in ameliorating key clinical parameters, including the WOMAC index, VAS scores, and high-sensitivity C-reactive protein levels in KOA patients. However, no statistically significant differences were observed in the BMI or knee joint rigidity. The evidence suggests that probiotic administration as an oral therapeutic intervention exhibits promising potential in KOA management through multiple mechanisms: symptomatic relief, analgesic effects, and modulation of inflammatory biomarkers. It should be noted that two studies within this meta-analysis implemented probiotic therapy as an adjunct to conventional pharmacological treatments (Wang, 2022; Han, 2023). Experimental animal models demonstrated that the synergistic administration of probiotics and chondroprotective agents exhibits enhanced therapeutic efficacy in patients with KOA, potentiating the probiotic-mediated anti-inflammatory response *in vivo* (Korotkyi et al., 2018; Korotkyi et al., 2020). Consequently, the administration of probiotics as an adjunctive therapy alongside conventional pharmaceutical interventions for KOA represents a novel therapeutic approach, potentially yielding enhanced clinical outcomes. Two studies within this investigation examined the impact of probiotic intervention on inflammatory biomarkers in KOA, analyzing multiple inflammatory mediators. The findings demonstrated that probiotic supplementation effectively downregulated the expression of pro-inflammatory cytokines, including interleukin-2 (IL-2), interleukin-6 (IL-6), interleukin-12 (IL-12), and tumor necrosis factor-alpha (TNF-*α*). This evidence suggests that probiotics can modulate inflammatory marker profiles in KOA patients, contributing to the amelioration of systemic inflammation and subsequently reducing synovial inflammation, joint effusion, and pain manifestations throughout KOA progression (Srivastava Rupesh, 2015; Muske and Knoop, 2023). Obesity represents a primary etiological factor in KOA (Shumnalieva et al., 2023). Despite this, two studies revealed no statistically significant differences in BMI changes between the probiotic intervention and control cohorts (Han, 2023; Dolatkhah et al., 2024). This may be attributed to the uncontrolled, multifaceted confounding variables impacting body weight across both investigations. Moreover, the sample sizes were inadequate, and the intervention durations were relatively short. Consequently, future research should consider extending the probiotic intervention period, increasing the sample size, and meticulously controlling various modifiable factors. It is also recommended to prolong the follow-up duration to thoroughly evaluate the effects of probiotics on BMI. While the efficacy of probiotics in obesity management remains contentious, substantial evidence suggests that probiotics play a crucial role in modulating lipid metabolism, demonstrating significant reductions in total cholesterol, low-density lipoprotein cholesterol, and triglyceride levels (Jia et al., 2021; Musazadeh et al., 2022; Zarezadeh et al., 2023). Therefore, extended probiotic administration demonstrates efficacy in obesity management. Notably, a singular study (Wang, 2022) examined bone mineral density (BMD) outcomes, revealing no significant differences between probiotic-supplemented KOA patients and controls. Nevertheless, substantial evidence from preclinical and clinical investigations indicates that probiotics positively influence bone metabolism and BMD, enhancing mineral bioavailability. This suggests their potential utility as an adjunctive therapy for BMD enhancement (Parvaneh et al., 2014). For example, a clinical investigation utilizing a randomized controlled trial methodology demonstrated that elderly female subjects with diagnosed osteoporosis exhibited attenuated BMD deterioration following *Lactobacillus reuteri* supplementation (Nilsson et al., 2018). Administration of *L. reuteri* demonstrated comparable outcomes in preclinical studies, wherein probiotic supplementation inhibited osteoclast formation and attenuated bone resorption in ovariectomy-induced osteoporotic mice, consequently mitigating skeletal deterioration (Britton et al., 2014). The findings indicate that probiotic supplementation demonstrates potential in modulating skeletal homeostasis and osteological protection through multiple interconnected pathways, encompassing gastrointestinal, neural, immunological, and endocrine regulatory mechanisms (Zhang Y. W. et al., 2023). Furthermore, probiotic supplementation demonstrates no adverse toxicological effects on human physiology, and optimal probiotic colonization enhances the homeostasis of the gut microbiota, thereby facilitating comprehensive systemic regulation of host physiological functions (Sanders et al., 2019). Consequently, the therapeutic approach should extend beyond mere probiotic supplementation, encompassing a comprehensive understanding of intestinal microbiota homeostasis and its pivotal role in both systemic health and osteoarthritis management. The maintenance of gut microenvironmental equilibrium can be primarily achieved through dietary modification and nutritional intervention strategies (Lozupone et al., 2012; Requena et al., 2018). Scientific evidence demonstrates that optimal nutritional practices significantly modulate inflammatory mediators, thereby exerting substantial influence on the pathophysiology of inflammatory musculoskeletal disorders (Dahan et al., 2017; Sureda et al., 2018). In addition, traditional Chinese medicine treatments, such as traditional Chinese medicines (Zhang et al., 2021), acupuncture (Wang et al., 2019; Jia et al., 2022) and exercise modulation (Zhang L. et al., 2023), have demonstrated potential in modulating gut microbiota composition. Nevertheless, the current body of evidence predominantly consists of preclinical investigations, with insufficient randomized controlled trials to establish clinical efficacy in osteoarthritis patients.

In addition, several limitations warrant consideration in this investigation: The limited sample size precluded a comprehensive assessment of publication bias, while substantial heterogeneity observed across analytical outcomes compromised the robustness of evidence supporting the measured endpoints. The assessment of clinical outcomes through WOMAC and VAS scoring systems presents inherent limitations due to their subjective nature, and the analysis would benefit from the incorporation of objective parameters, including biochemical markers and radiological findings. The limited selection of probiotic strains investigated in this study, specifically *S. boulardii*, TCI633, *Lactobacillus casei* Shirota (LcS), and *Bifidobacterium bifidum*, represents only a fraction of the diverse probiotic species available for therapeutic applications. This narrow scope necessitates further comprehensive investigations encompassing a broader spectrum of probiotic strains to establish their therapeutic potential in KOA management. The investigation demonstrated substantial variability in oral probiotic administration protocols, necessitating additional clinical trials to elucidate the potential differential impacts of diverse probiotic dosing schedules and concentrations on therapeutic outcomes in KOA patients.

## Data Availability

The original contributions presented in the study are included in the article/supplementary material, further inquiries can be directed to the corresponding authors.
